# COVID-19: Global Health Equity in Pandemic Response

**DOI:** 10.4269/ajtmh.20-0260

**Published:** 2020-04-14

**Authors:** Louise C. Ivers, David A. Walton

**Affiliations:** 1 Center for Global Health, Massachusetts General Hospital, Boston, Massachusetts;; 2 Department of Global Health and Social Medicine, Harvard Medical School, Boston, Massachusetts;; 3 Division of Global Health Equity, Brigham and Women’s Hospital, Boston, Massachusetts

As the world struggles with the rapidly evolving pandemic of novel coronavirus disease (COVID-19), evidence and experience suggest that low-income and marginalized communities in our global society will bear the biggest impact. We know this because, with our colleagues in Boston, Haiti, Uganda, and Sierra Leone, we have worked in under-resourced, overstretched, and overwhelmed health systems for our whole careers. We know we will see the devastating impact of this pandemic on those who are already marginalized; COVID-19 will amplify existing inequities, and we must act swiftly to leave no one behind.

Diagnostics, pharmaceutical interventions, and public health solutions for other pandemic diseases such as tuberculosis, HIV infection, and cholera have never fully “trickled down” to marginalized or impoverished communities. That is why, for example, HIV infection rates remain alarmingly high among adolescent girls in South Africa,^1^ and transgender people and communities of color in the United States,^2^ and why tuberculosis still remains a major public health concern despite the advent of effective therapy for drug-sensitive disease almost 50 years ago.^3^ As the world races to find an effective antiviral against severe acute respiratory syndrome coronavirus-2, as well as a vaccine, will these become sovereign commodities of the global North? We see no reason why injustices of the past will not be repeated just because the pathogen is novel. Often, widespread access to lifesaving therapeutics—like treatment for HIV infection—has only been made possible for marginalized groups through hard-fought activism. But activism alone is not enough—in 2019, the U.S. government declined influenza vaccinations to detainees of the Customs and Border Protection agency despite documented deaths of children in these detention facilities from influenza and protests by medical professionals.^4^

As the incidence of COVID-19 escalates in low-income countries, we have five specific concerns as global health faculty at large U.S. teaching hospitals. First, it is well documented that structural and institutional racism, and the marginalization of migrant communities, perpetuate differential outcomes in health. Poor or otherwise marginalized communities, including communities of color, have been systematically left behind as biomedical progress advances. Existing health disparities, such as disparities in the prevalence of pulmonary disease, highlight worrisome risk factors for increased incidence of COVID-19. Asthma rates are higher among U.S. black and native American children than U.S. white children, and asthma is most prevalent in the poorest socioeconomic groups.^5^ Tobacco companies, after spending decades enticing the use of their products in low-resourced settings, have acquired 1.1 billion customers who smoke, of whom 80% reside in low- and middle-income countries.^6^ Long-term consequences of smoking, such as chronic lung disease, have already been linked to worse COVID-19 outcomes. As of this date, in mid-April 2020, most confirmed cases of COVID-19 have been in Europe, East Asia, and the United States, places where noncommunicable diseases predominate the health landscape. We know very little about how malnutrition, tuberculosis, HIV infection, and soil-transmitted helminth infections will impact the disease course of COVID-19, but these could have major implications for low-resourced communities in which the prevalence of these diseases is high.

Second, although almost every health system’s capacity would be surpassed by an unmitigated surge in COVID-19 cases, fragile health systems in low-income settings struggle daily to diagnose and treat even moderately unwell people because of chronic shortages of trained staff, effective therapeutics, diagnostics, and built infrastructure. Global inequity in maternal mortality, under-5 diarrheal mortality, and deaths from pneumonia all highlight this fact. Already, around the world, there are countless unnecessary deaths on a daily basis—“senseless deaths” that never needed to happen because we know what to do. We have just failed to deliver.

Material deprivations in the built health environment are pervasive, and personal protective equipment to care for patients with transmissible diseases are already in desperately low supply in many countries. Laboratory capacity is often limited by a lack of staff and supplies, and laboratories are frequently centralized in urban regions, leaving rural places without access. Low testing rates for COVID-19 in countries such as Haiti, Yemen, and Central African Republic emphasize already how challenging it will be to even understand the pandemic without accessible diagnostics; never mind trace and isolate those people with infection for effective control.

Although the year 2020 opened with a celebration of the international year of the nurse and midwife, the WHO’s most recent data estimated a global deficit of 17.4 million health workers, mainly in Africa and Southeast Asia.^7^ Although our own hospitals in Boston scramble to create rosters with three or four layers of staffing backup to cope with forecasted numbers of sick or exposed staff and patient flow, how will communities with a drastically insufficient health workforce at baseline cope?

Third, in many circumstances, it is impossible to quarantine at home or to self-isolate. Homeless people, displaced populations, and prisoners cannot choose to be physically distant from others. Haiti, where we have worked for almost two decades, is one of the most densely populated countries in the Latin America Caribbean region, with whole families often living in humble one- or two-room abodes. Social distancing, one key part of aggressive containment strategies, is not feasible for large swathes of the world, and advisories to stay at home ignore the realities of some of the 820 million food insecure people in the world, for whom the day’s food is acquired only at the end of the day’s work. Food insecurity has a detrimental impact on health which is why, for example, in the midst of a major epidemic in Haiti in 2012, cholera was almost twice as deadly for families that were severely food insecure than for families with steady access to food.^8^

Fourth, water insecurity and lack of access to safe sanitation and hygiene will undermine a basic pillar of COVID-19 prevention in many parts of the world. Twenty-nine percent of the global population lacks safely managed drinking water, and three billion people do not have access to soap or water for handwashing at home.^9^ The United States offers no exception, as the prevalence of exposure to hookworm in a rural Alabama community and the failure to realize the right to clean water in Flint, Michigan, both demonstrate.^10,11^

Fifth, although the direct impact of COVID-19 is self-evident, the indirect impact of the pandemic on other health indicators may be less visible. Fragile health systems that are already stretched have little to no elasticity in supply and are at risk for the biggest impacts of these indirect effects. The 2014 Ebola virus disease epidemic in West Africa, for example, resulted in more than 4,000 maternal deaths, 6,700 infant mortalities, and approximately 3.5 million more cases of untreated malaria because the health system effort was, by necessity, redirected to the viral epidemic response.^12^

What does global health equity mean in a pandemic of such magnitude as COVID-19? It means we must take a proactive approach, addressing disparities and injustice from the start—both in how we approach scientific discovery and in how we deliver interventions.

Scientists, practitioners, and community leaders in low-resource communities have innovations, hypotheses, and proposed solutions to the COVID-19 pandemic. The global public health elite must have the humility to include these as part of collective action. We must commit, as a global community, to ensuring that when pharmaceutical interventions are proven, they will be accessible to all, not only those who can afford to pay. We need COVID-19 incidence and mortality data that are disaggregated by gender, race, and location. We need radical social investments to support the most impoverished, and we must decongest prisons and release detained asylum seekers to prevent unnecessary deaths. Multilateral investment in health systems strengthening as a fundamental principle of global health equity has never been more important than now. With an ambitious belief in what it is possible to do for the poorest communities, we must commit to global health equity, leaving no one behind.
